# Student evaluation of an OSCE in paediatrics at the University of the West Indies, Jamaica

**DOI:** 10.1186/1472-6920-4-22

**Published:** 2004-10-16

**Authors:** Russell B Pierre, Andrea Wierenga, Michelle Barton, J Michael Branday, Celia DC Christie

**Affiliations:** 1 Department of Obstetrics, Gynaecology and Child Health, University of the West Indies, Jamaica; 2 Undergraduate Affairs, Dean's Office, Faculty of Medical Sciences, Jamaica

## Abstract

**Background:**

The Faculty of Medical Sciences, University of the West Indies first implemented the Objective Structured Clinical Examination (OSCE) in the final MB Examination in Medicine and Therapeutics during the 2000–2001 academic year. Simultaneously, the Child Health Department initiated faculty and student training, and instituted the OSCE as an assessment instrument during the Child Health (Paediatric) clerkship in year 5. The study set out to explore student acceptance of the OSCE as part of an evaluation of the Child Health clerkship.

**Methods:**

A self-administered questionnaire was completed by successive groups of students immediately after the OSCE at the end of each clerkship rotation. Main outcome measures were student perception of examination attributes, which included the quality of instructions and organisation, the quality of performance, authenticity and transparency of the process, and usefulness of the OSCE as an assessment instrument compared to other formats.

**Results:**

There was overwhelming acceptance of the OSCE in Child Health with respect to the comprehensiveness (90%), transparency (87%), fairness (70%) and authenticity of the required tasks (58–78%). However, students felt that it was a strong anxiety-producing experience. And concerns were expressed regarding the ambiguity of some questions and inadequacy of time for expected tasks.

**Conclusion:**

Student feedback was invaluable in influencing faculty teaching, curriculum direction and appreciation of student opinion. Further psychometric evaluation will strengthen the development of the OSCE.

## Background

The assessment of student's clinical competence is of paramount importance, and there are several means of evaluating student performance in medical examinations [1,2]. The Objective Structured Clinical Examination (OSCE) is an approach to student assessment in which aspects of clinical competence are evaluated in a comprehensive, consistent and structured manner, with close attention to the objectivity of the process [3]. The OSCE was introduced by Harden in 1975 [4], and first described as an assessment format in Paediatrics (Child Health) by Waterson and colleagues [5]. Since its inception, the OSCE has been increasingly used to provide formative and summative assessment in various medical disciplines worldwide [6], including non-clinical disciplines [7].

The University of the West Indies was established in 1948 as a medical college of the University of London, which granted external degrees to those who successfully completed the course [8]. The Faculty of Medical Sciences located on four campuses, on the islands of Jamaica, Bahamas, Barbados and Trinidad and Tobago, conducts bi-annual final examinations at the end of year 5. The 'traditional' format of examination that included long case, short cases and oral examination, was preserved until recent changes in the curriculum. In response to recommendations to improve the validity and fairness of the examination through adoption of proven methods and approaches in assessment and evaluation in medical education, the Faculty of Medical Sciences (FMS), University of the West Indies (UWI) initiated the OSCE as a formal method of assessment for the final examination in Medicine and Therapeutics, Child Health, Community Health and Psychiatry, in November 2000. Students and faculty were exposed for the first time to a relatively new assessment instrument in which aspects of competence (communication, history-taking and technical skills) were assessed in a structured, formal manner.

The Section of Child Health, Mona, Jamaica, implemented the OSCE examination as an end-of clerkship assessment for students in their 5^th ^year, during the 1999–2000 academic year. It was felt timely in order to (a) direct and motivate student learning in areas not previously assessed in the 'traditional' curriculum, (b) verify students' competence in fundamental paediatric clinical skills, and (c) provide a forum for feedback to students on their strengths and weaknesses in clinical skills. It was thought that it would enhance faculty and student acceptance of this new assessment tool and promote faculty training for the newly introduced final OSCE examination.

In the absence of any previous information from this institution, the study was designed to evaluate student overall perception of the end-of-clerkship OSCE, determine student acceptability of the process and provide feedback to enhance further development of the assessment.

## Methods

**Figure 1:**
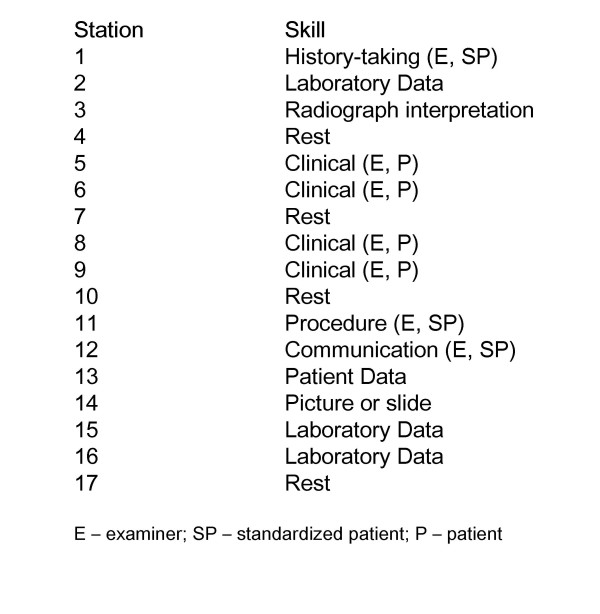
Plan of OSCE circuit

The OSCE comprised a circuit of thirteen stations, which involved completion of a number of tasks such as examination of a system, eliciting a focussed history, counselling or communicating a problem, performing a procedure and problem-solving oriented around patient and laboratory data, and photographic material (Figure 1). The areas assessed included cardiovascular, respiratory, abdomen, neurological, developmental, dysmorphism and nutrition. This assessment format allowed the controlled exposure of students to a wide variety of paediatric clinical skills within a relatively short time period. Each station was 7 minutes duration with the exception of the 14-minute history-taking station. One minute was given between stations to facilitate change and the reading of instructions. With the inclusion of strategically placed rest stations, to reduce student and patient fatigue, all students completed the circuit over a 2-hour period.

A standardised technique of marking was used and student performance was assessed by criterion reference for each station. Criterion-based scoring was used, with each checklist item scored as 0 (omitted, incorrect or inadequate), or 1–2 (correct or adequate).

Face and content validity of each checklist was established by review and consensus by a core group of senior paediatricians. Stations were first selected to represent the curricular goals and objectives and to reflect authentic clinical situations. Checklists were designed to include the features thought to be most important by the development committee. Through discussions, consensus was achieved on the checklist items and structure.

The study was conducted during the period July 2001 to December 2002. Five groups of students participated in the process, during their respective clerkship rotations. Student groups had at least two briefing sessions before the OSCE, and included an orientation about the examination process (both end-of-clerkship and final MB) and a review of commonly assessed competences. They were also apprised of the valuable contribution they could make towards improving the assessment and encouraged to participate in the evaluation.

A cross-sectional survey using a 32-item self-administered questionnaire was completed at the end of each OSCE [9]. Students were asked to evaluate the content, structure, and organization of the OSCE, rate the quality of performance and objectivity of the OSCE process, and to give their opinion about the usefulness of the OSCE as an assessment instrument compared to other forms which they had experienced (essays, multiple choice questions, long and short cases, general clerkship rating).

Participation was on a voluntary basis and students were assured that those who declined involvement in the survey would not be penalised. The Curricular Affairs Section handled the administration and analysis of the questionnaires. Ethical approval was received from the University Hospital of the West Indies/University of the West Indies Faculty of Medical Sciences Ethics Committee. Following completion of the questionnaire, an OSCE review session was conducted with the students for feedback and teaching purposes, at the end of the clerkship. Students were given the opportunity to review their individual performances at the respective stations. Examiner evaluations were also used in the feedback process.

Data were collated and descriptive and non-parametric tests applied using Stata version7 [10]. Basic statistical analysis of the Likert items was conducted by calculating frequencies, means and standard deviations. Qualitative analysis was done through a form of content analysis by identifying themes in student responses and grouping responses according to thematic content. Two of the authors individually conducted this content analysis and identified themes and final grouping of responses were developed by consensus.

## Results

### OSCE evaluation

Eighty-one students responded to the questionnaire, representing 92 % (81/88) of those who completed the Clerkship.

The majority of students agreed that the OSCE was comprehensive and covered a wide range of knowledge (95%) and clinical competencies (86%) in Child Health. Three quarters (78%) also agreed that the assessment process helped to identify weaknesses and gaps in their competencies (Table 1).

**Table 1:** OSCE evaluation

Question	Agree %	Neutral %	Disagree %	No comment %
Exam was fair	68	19	12	1
Wide knowledge area covered	95	5		
Needed more time at stations	70	22.5	7.5	
Exams well administered	73	16	11	
Exams very stressful	67	20	13	
Exams well structured & sequenced	81.5	17	2.5	
Exam minimized chance of failing	28	40.5	30	1.5
OSCE less stressful than other exams	15	40	35	10
Allowed student to compensate in some areas	67	21	12	
Highlighted areas of weakness	78	13	9	
Exam intimidating	48	32	20	
Student aware of level of information needed	53	26	21	
Wide range of clinical skills covered	86	6	8	

Most (73–82%) felt that the exam was well administered, and that the stations were arranged in an organised and well-sequenced order.

Students believed that the assessment was fair (68%). Fifty-three percent were aware of the level of information required at each station, yet 28% felt that the examination process minimized their chances of failing.

Students found the OSCE to be intimidating (48%) and more stressful (35%) than other assessment formats to which they were previously exposed. And most (70%) felt that they needed more time to complete the stations.

### Performance testing

The majority of students felt they were well oriented about the exam and that the required tasks were consistent with the actual curriculum that they were taught. They also felt that the process was fair but were not as satisfied with the time allocation for each station (Table 2).

**Table 2:** Quality of performance testing

Question	Not at all %	Neutral %	To great extent %
Fully aware of nature of exam	4	9	87
Tasks reflected those taught	4	23	73
Time at each station was adequate	44	35	21
Setting and context at each station felt authentic	18	24	58
Instructions were clear and unambiguous	15	27	58
Tasks asked to perform were fair	3	27	70
Sequence of stations logical and appropriate	13	30	57
Exam provided opportunities to learn	11	21	69

Most saw the OSCE as a useful learning experience and that the content reflected real life situations in Child Health. More than half of the students were satisfied with the conduct, organisation and administration of the OSCE.

### Perception of validity and reliability

Although half of the students believed that the scores were standardised, they were unsure whether their scores were an actual reflection of their paediatric clinical skills (Table 3). Student responses to the question about bias due to gender, personality or ethnicity, were not interpretable.

**Table 3:** Student perception of validity and reliability

Question	Not at all %	Neutral %	To great extent %
OSCE exam scores provide true measure of essential clinical skills in paediatrics	14	43	43
OSCE scores are standardized	8	37	55
OSCE practical and useful experience	4	23	73
Personality, ethnicity and gender will not affect OSCE scores	18	19	63

### Comparing assessment formats

Students were asked to rate the following assessment instruments to which they had been exposed (multiple choice questions, essays / short answer questions, general clerkship ratings, OSCE). A likert scale was used to assess each according to the evaluative labels (Table 4).

**Table 4:** Student rating of assessment formats

Question:	Difficult %	Undecided %	Easy %
**Which of the following formats is easiest?**			
MCQ	48	26	26
Essay/SAQ	38	44	18
OSCE	43	45	12
Clerkship ratings	21	47	32

Question:	Unfair %	Undecided %	Fair %

**Which of the following formats is fairest?**			
MCQ	29	28	43
Essay/SAQ	7	25	68
OSCE	4	16	80
Clerkship ratings	16	26	58

Question:	Learn very little %	Undecided %	Learn a lot %

**From which of the following formats do you learn most?**			
MCQ	28	37	35
Essay/SAQ	12	37	51
OSCE	15	25	60
Clerkship ratings	20	18	62

Question:	Used much less %	Undecided %	Used much more %

**Which of the following formats should be used more often in the clinical years of the programme?**			
MCQ	31	59	10
Essay/SAQ	9	52	39
OSCE	5	43	52
Clerkship ratings	12	56	32

MCQ – multiple choice question; SAQ – short answer question; OSCE -objective structured clinical examination

Thirty-two percent of students felt that the clerkship rating was the easiest, while 48% rated MCQ as a more difficult form of assessment. The OSCE was overwhelmingly considered the fairest assessment format (80%), and essays (68%) to a lesser extent. OSCE (60%) and clerkship ratings (62%) were considered the most useful learning experiences. Compared to the other assessment formats, 52% considered that the OSCE should be used most in the clinical years.

### Qualitative data

Students were asked follow-up questions related to positive and negative aspects of the OSCE and suggestions for improvement. The open-ended responses were grouped by thematic content.

Among the positive attributes of the OSCE, students re-affirmed that the assessment was comprehensive (44 comments) and that it was an objective and fair process (43 comments). Some indicated that the opportunity for feedback helped to motivate them and drive the learning process (21 comments).

Students felt that the time allocated to perform expected tasks was insufficient (36 comments), and that the procedure was stressful (18 comments) and tiring (13 comments). Technical problems (28 comments) included unclear instructions, inadequate time provision and instructions between stations and detention of some candidates at stations by examiners.

Suggestions for improvement included increasing the duration of stations (29 comments), ensuring clear instructions (8 comments) and having more realistic expectations of students for the expected tasks. A few students wished to have more training with the OSCE and suggested that the examination should be videotaped to increase objectivity and permit review.

## Discussion

Students overwhelmingly perceived that the OSCE in Child Health had good construct validity. This was demonstrated by the favourable responses concerning transparency and fairness of the examination process, and the authenticity of the required tasks per station. Excellent levels of acceptance of the OSCE by students have been previously described in the literature [11-14]. They however expressed concerns and uncertainty about whether the process would minimize their chances of failing or that the results were a true reflection of their clinical skills. This was understandable, since it was their first encounter with this type of assessment.

Several felt that the examination was stressful and intimidating, yet paradoxically some students perceived it as an enjoyable, practical experience. Studies surveying student attitudes during the OSCE have documented that the OSCE can be a strong anxiety-producing experience, and that the level of anxiety changes little as students progress through the examination [15].

It is well recognised that assessment is a catalyst for both curriculum change and student learning. The students recognised the value of the instrument for formative evaluation. In addition, as many medical schools have adopted a student-centred approach to medical education, greater student participation in quality assurance exercises must be encouraged. Students perceived the OSCE to be fairer than any other assessment format to which they were exposed. The findings were somewhat similar to the views of students at Newcastle medical school [16]. Although student views on fairness may not be consistent with published literature, the impact and influence on acceptability of the instrument should be noted.

They offered constructive criticism of the structure and organisation of the process. At some stations they felt that the instructions were ambiguous and that the time allocation was inadequate for the expected tasks. The feedback was invaluable and facilitated a critical review and modification of the station content and conduct of the examination over time. Faculty perceived that the concerns about time allocation per station and the degree of stress expressed by the students were due to inadequate preparation for the examination, particularly in competences not previously assessed in the 'traditional' examination.

The high student response rate has helped to ensure that the findings presented are a valid representation of student opinion. Students have traditionally viewed the end-of-clerkship assessment as a 'high-stake' examination and also perceive it as predictive of their performance at their final MB examination. Student perception of the OSCE however, may have been influenced by anxiety and lack of confidence associated with a new assessment. The responses may also have been affected by the timing of the inquiry (immediately after the examination); hence student stress and fatigue should be taken into consideration. Whereas the high response rate ensured that the views were reasonable representative of the students, differences in assessors could have influenced the interpretation of the results of open-ended responses.

Implementing the OSCE in Child Health at the University of the West Indies, Jamaica has been challenging, however student participation in the evaluation and their overall acceptance of the instrument have been encouraging. Feedback from students and faculty has been useful in effecting improvements to the process and greater emphasis has been placed on the teaching and evaluation of history taking, communication and technical competencies. It is also sending a clear message to students that the achievement of overall competence is imperative to clinical practice in the current environment. Ultimately, these provide the loop necessary to drive the continuum of curriculum development. This has been timely considering that the Faculty of Medical Sciences, Jamaica is undergoing significant reform [17]. Further developments involving psychometric evaluation will strengthen the process.

## Conclusions

In summary, the findings highlight the need for student participation in the development of new assessment tools in medical curricula. Student acceptance will be more favourable for assessment formats that they perceive to be transparent, authentic and valid. 'Traditional' medical curricula must be responsive to global paradigm shifts in undergraduate medical education.

## Competing interests

The authors declare that they have no competing interests.

## Authors' contributions

RP conceptualised the study; developed the proposal, coordinated the conduct of the project, completed initial data entry and analysis, and wrote the report. AW participated in the design of the study, coordinated the conduct of the project, performed the statistical analysis, and assisted in writing the report. MB was the main organizer of the clerkship OSCE, and assisted in editing the final report. MBr and CC participated in overall supervision of project and revision of report. All authors read and approved the final manuscript.

## Pre-publication history

The pre-publication history for this paper can be accessed here:



## References

[B1] Harden RM (1979). How to assess clinical competence – an overview. Med Teach.

[B2] Fowell SL, Bligh JG (1998). Recent developments in assessing medical students. Postgrad Med J.

[B3] Harden RM (1988). What is an OSCE?. Med Teach.

[B4] Harden RM, Stevenson M, Downie WW, Wilson GM (1975). Assessment of clinical competence using objective structured examination. Br Med J.

[B5] Waterson T, Cater JI, Mitchell RG (1980). An objective undergraduate clinical examination in child health. Arch Dis Child.

[B6] Carraccio C, Englander R (2000). The objective structured clinical examination, a step in the direction of competency-based evaluation. Arch Pediatr Adolesc Med.

[B7] Harden RM, Caincross RG (1980). The assessment of practical skills: the Objective Structured Practical Examination (OSPE). Stud High Educ.

[B8] Sherlock P, Nettleford R (1990). The University of the West Indies: a Caribbean response to the challenge of change.

[B9] De Lisle J (2001). 2001 Phase 2, OSCE student evaluation form.

[B10] StataCorp (2001). Stata Statistical Software: Release 70.

[B11] Newble DI (1988). Eight years experience with a structured clinical examination. Med Educ.

[B12] Duerson MC, Romrell LJ, Stevens CB (2000). Impacting faculty teaching and student performance: nine years' experience with the objective structured clinical examination. Teach Learn Med.

[B13] Kowlowitz V, Hoole AJ, Sloane PD (1991). Implementation of the Objective Structured Clinical Examination in a traditional medical school. Acad Med.

[B14] Woodburn J, Sutcliffe N (1996). The reliability, validity and evaluation of the objective structured clinical examination in podiatry. Assessment Evaluation Higher Educ.

[B15] Allen R, Heard J, Savidge M, Bittengle J, Cantrell M, Huffmaster T (1998). Surveying students' attitudes during the OSCE. Adv Health Sci Educ.

[B16] Duffield KE, Spencer JA (2002). A survey of medical students' views about the purposes and fairness of assessment. Med Educ.

[B17] (2003). Faculty of Medical Sciences, University of the West Indies, Mona. Report – MB, BS Undergraduate Programme, First Annual Report on Curriculum Development.

